# Recurrent pregnancy loss and fetal complete heart block as the initial manifestations of maternal systemic lupus erythematosus: a case report on the diagnostic and preventive role of anti-SSA (Ro) antibodies

**DOI:** 10.1186/s12884-025-07820-9

**Published:** 2025-07-09

**Authors:** Ali Akbari, Fatemeh Palizvan, Arvin Mirshahi, Forod Salehi

**Affiliations:** 1 Students’ Scientific Research Center, Tehran University of Medical Sciences, Tehran, Iran; 2 School of Medicine, Abadan University of Medical Sciences, Abadan, Iran; 3 Department of Cardiology, Cardiovascular Diseases Research Center, Birjand University of Medical Sciences, Birjand, Iran

**Keywords:** Systemic lupus erythematosus, Anti-SSA (Ro), Complete heart block, Case report

## Abstract

**Background:**

Systemic Lupus Erythematosus (SLE) is a chronic autoimmune disease that causes multi-organ damage and primarily affects women of reproductive age. Although pregnancy in these patients carries increased risks, advances in management have significantly improved outcomes for both the mother and the fetus.

**Case presentation:**

A 32-year-old woman with a history of two stillbirths and an infant death due to a complete heart block (CHB) was referred at 17 weeks of gestation for fetal echocardiography, which showed no abnormalities. Further clinical evaluation revealed systemic features including painless mucosal ulcers, intermittent synovitis, and mild pericardial effusion. Positive anti-SSA (Ro) antibodies and a positive ANA test (1:80, homogeneous pattern) supported the classification of SLE. Hydroxychloroquine (HCQ) treatment was started, and follow-up echocardiograms revealed normal fetal heart function. The pregnancy progressed without complications, resulting in the birth of a healthy baby with normal cardiac findings.

**Conclusion:**

The diagnosis of SLE in this case was based on a combination of clinical manifestations and immunologic findings, in accordance with the ACR/EULAR 2019 criteria. While HCQ may have contributed to the favorable fetal outcome, spontaneous improvement or other modifying factors cannot be excluded. Early maternal assessment and timely initiation of treatment remain critical for optimizing outcomes in high-risk pregnancies.

**Supplementary Information:**

The online version contains supplementary material available at 10.1186/s12884-025-07820-9.

## Background

Systemic Lupus Erythematosus (SLE) is a chronic autoimmune disease and a multi-organ disorder characterized by the dysregulation of T and B cells. This autoimmune process leads to the production of pathogenic autoantibodies, ultimately resulting in the formation and deposition of immune complexes in various tissues throughout the body. The deposition of these immune complexes triggers inflammation and tissue damage in multiple organs, including the skin, joints, kidneys, central nervous system, and cardiovascular system [1].

Most patients with SLE have a positive antinuclear antibody (ANA) test, and other autoantibodies such as anti-dsDNA and anti-Sm are also often present. The diagnosis of the disease requires a positive ANA test along with clinical manifestations or the presence of other autoantibodies [2].

SLE primarily affects women of reproductive age, and as such, fertility and pregnancy-related issues play a significant role in the care of these patients. Pregnancy in women with SLE is associated with an increased risk of disease flare and adverse pregnancy outcomes, including preeclampsia, preterm birth, intrauterine growth restriction (IUGR), and neonatal risks such as neonatal lupus erythematosus (NLE) and complete heart block (CHB) [3, 4].

Previously, these factors, coupled with limited knowledge, led many physicians to discourage their SLE patients from pursuing pregnancy. However, over the past few decades, a better understanding of SLE and its management during pregnancy has improved maternal and fetal outcomes. The evaluation of this case is of particular importance as SLE and its associated complications, especially during pregnancy, can have serious consequences for both the mother and the fetus. Accurate assessment and appropriate management play a crucial role in mitigating these complications.

This case report has been prepared according to the CARE (CAse REport) guidelines for case reports [5].

## Case presentation

**Fig. 1:**
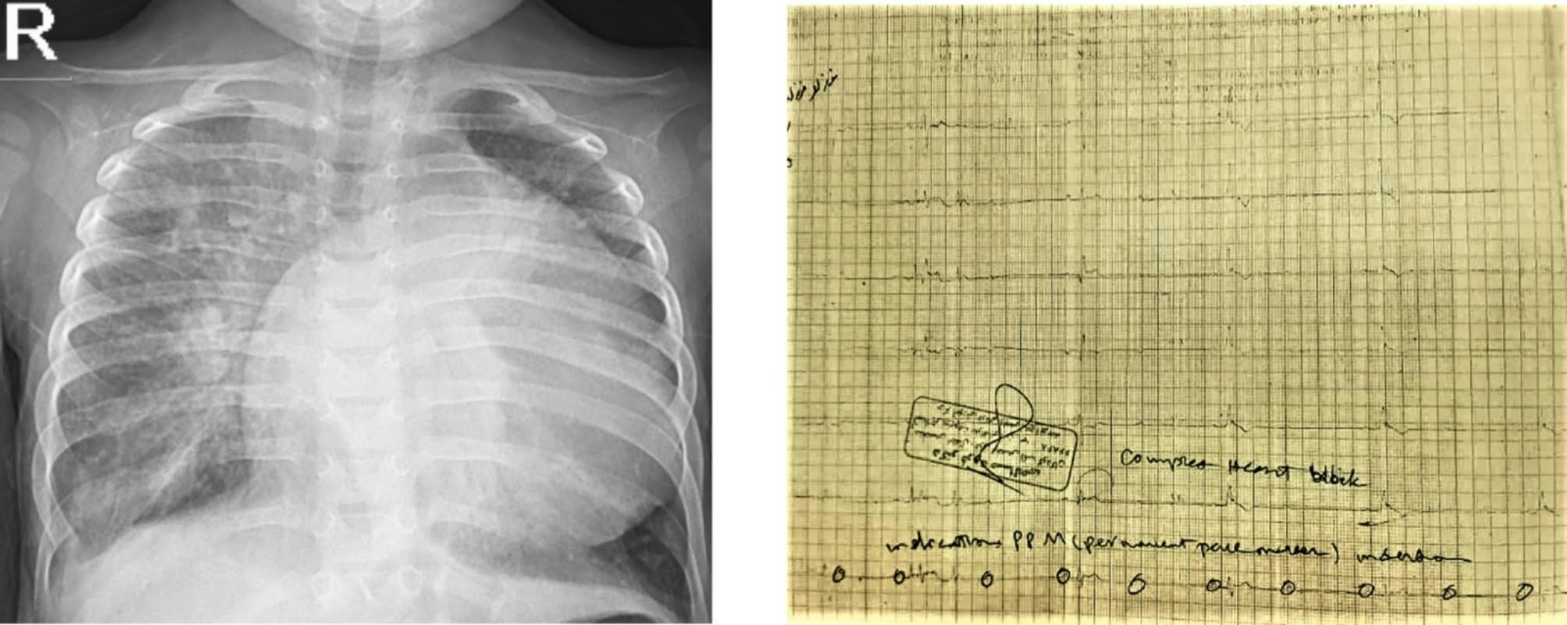
Chest X-ray and 12-lead ECG of a dead neonate with congestive heart failure and CHB, born to a mother with pre-diagnosed SLE

A 32-year-old woman presented with a history of two stillbirths occurring beyond 20 weeks of gestation and one live birth three years ago. The live-born infant was diagnosed at birth with complete (third-degree) heart block, congestive heart failure (severe cardiomegaly), pleural effusion, and abdominal ascites, and unfortunately passed away 48 h postpartum (Fig. 1).

At the time of the neonatal death due to complete heart block (CHB), no immunologic testing, including anti-SSA antibody, was performed. Although the patient had experienced occasional painless oral and nasal ulcers as well as joint swelling in the left wrist and right hand, these symptoms were not evaluated further at that time. No rheumatologic referral was made, and the underlying autoimmune condition remained undiagnosed until the current pregnancy.

The patient, currently at 17 weeks of gestation, was referred for fetal echocardiography due to her medical history and suspicion of SLE based on the previous infant diagnosed with CHB. No pathological cardiac abnormalities were observed on fetal Doppler echocardiography.

The fetal heart rate and rhythm were reported as normal, with a heart rate of 154 beats per minute and a left ventricular ejection fraction (EF) of 60%. Given the patient’s history of two stillbirths, the birth of an infant with complete heart block, and suspicion of SLE, the attending physician had ordered specialized immunologic tests to evaluate for SLE. Given the patient’s history of two stillbirths, the birth of an infant with complete heart block, and suspicion of SLE, the attending physician has ordered specialized immunological tests to evaluate the presence of SLE.

**Fig. 2:**
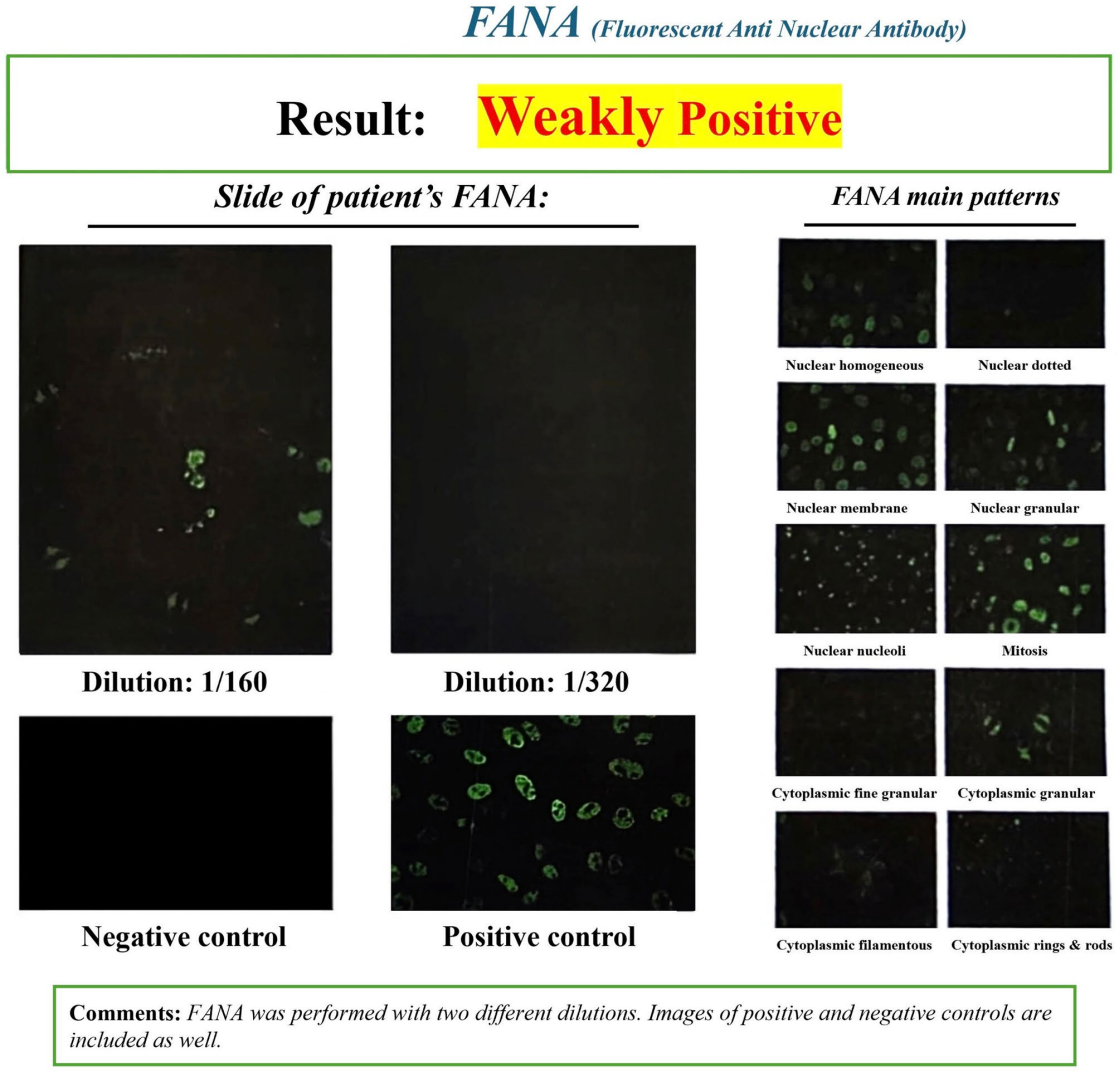
Weakly positive FANA test result

The results of the patient’s specialized immunological tests were reported as negative (Table 1). In the patient’s ANA profile, SS-A native (60 kDa) was reported as positive (Table 2). The patient’s FANA test was also reported as weakly positive (Fig. 2). The ANA titer was 1:80, and the test was performed using indirect immunofluorescence (IIF) on HEp-2 cells. The observed fluorescence pattern was homogeneous.

**Table 1:** Results of immunological tests

Test	Result	Unite
serology		mg/L
CRP Quantitive	7.32	
Immunoassays-Autoimmune Diseases		
Anti lupus Anticoagulant IgG-Screen	Neg (0.97)	
Anti ds-DNA	Neg (0.1)	
Anti Cardiolipin (IgG)	Neg (1.78)	
Anti Cardiolipin (IgM)	Neg (2.69)	

**Table 2:** Positive SS-A native (60 kDa) in the ANA profile (Western Blot)

Specific Antibody	Intensity	Result	Explanation
Mi-2	2	**-**	Negative
Ku	2	**-**	Negative
RNP/Sm	0	**-**	Negative
Sm	1	**-**	Negative
SS-A native (60 kDa)	14	**+**	Positive
Ro-52 recombinant	4	**-**	Negative
SS-B	1	**-**	Negative
Scl-70	0	**-**	Negative
PM/Scl100	0	**-**	Negative
Jo-1	2	**-**	Negative
centromere B	1	**-**	Negative
PCNA	0	**-**	Negative
dsDNA	1	**-**	Negative
Nucleosomes	2	**-**	Negative
Histones	2	**-**	Negative
Ribosomal Proteins	3	**-**	Negative
AMA-M2	1	**-**	Negative

Given the positive Anti-SSA (Ro) Ab and the history of a newborn with complete heart block, the diagnosis of SLE was confirmed in this patient. Further clinical evaluation revealed painless oral and nasal ulcers, synovitis affecting the left wrist and the second and third metacarpophalangeal joints of the right hand, and a mild pericardial effusion on echocardiography. ANA was positive at a titer of 1:80, which satisfies the entry criterion for SLE classification. According to the 2019 ACR/EULAR classification criteria, the patient scored 1 point each for oral/nasal ulcers, synovitis, and serositis, resulting in a total of 4 points—meeting the minimum threshold for SLE diagnosis. Consequently, with the diagnosis of SLE during pregnancy at 17 weeks, treatment with hydroxychloroquine (200 mg daily) was initiated, and the patient was advised to undergo follow-up echocardiograms. Hydroxychloroquine was initiated at a dose of 200 mg daily, which falls within the generally recommended range of 200–400 mg/day for SLE treatment. The dose selection was based on clinical judgment and safety considerations, as there is no specific weight-based guideline for hydroxychloroquine dosing during pregnancy in standard references. Repeated echocardiograms at weeks 23 and 27 showed no pathological cardiac abnormalities. The fetal heart rate and rhythm were normal, with heart rates of 158 and 140 beats per minute, and the left ventricular EF was reported as 57% and 76%, respectively.

**Fig. 3:**
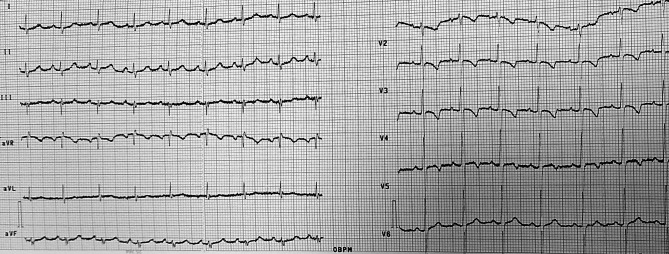
12-lead ECG of a healthy 3-month-old infant, after the mother’s treatment with HCQ

Due to the early diagnosis of SLE at 17 weeks of gestation and the initiation of hydroxychloroquine treatment, the fetal cardiac function remained normal throughout the pregnancy. The pregnancy progressed without complications, and a healthy baby was born with normal echocardiography and electrocardiography findings (Fig. 3).

## Discussion and conclusion

The diagnosis of SLE is based on a combination of clinical symptoms and laboratory results. Antinuclear antibodies (ANA) are typically positive in over 95% of patients and are used as an initial screening marker among immunological tests. Additionally, anti-dsDNA and anti-Sm antibodies are more specifically associated with SLE, and their positivity, particularly in the presence of clinical symptoms, is crucial for confirming the diagnosis. In certain cases, the presence of specific antibodies, such as Anti-SSA (Ro) and Anti-SSB (La), plays a significant role in providing a more accurate diagnosis and predicting pregnancy-related complications [6].

A notable point in this case is the positivity of only one immunological test, Anti-SSA (Ro), which was insufficient for a definitive diagnosis of SLE. However, the diagnosis was confirmed based on the combination of this laboratory finding and the patient’s clinical history, which included two previous miscarriages and the birth of a neonate with a complete heart block. This case highlights that the diagnosis of SLE cannot rely solely on laboratory findings or patient history in isolation. A comprehensive assessment that includes both systemic clinical features and immunologic evidence is essential to support a diagnosis in accordance with established classification criteria [7].

SLE is associated with a high risk of fetal complications. NLE is a rare acquired autoimmune condition characterized by dermatologic, cardiac, and systemic abnormalities in newborns whose mothers have antibodies against the autoantigens type A (Ro/SSA) or type B (La/SSB) [8].

The underlying mechanism of NLE involves the passive transfer of maternal autoantibodies targeting antigenic components within the ribonucleoprotein complex (60 kDa Ro, 52 kDa Ro, 48 kDa La) across the placenta [9].

Common cardiovascular manifestations include bradycardia, prolonged QT intervals, cardiomyopathy, congestive heart failure, myocarditis, and structural anomalies such as ventricular septal defects (VSD), atrial septal defects (ASD), patent foramen ovale (PFO), patent ductus arteriosus (PDA), and pulmonary stenosis [10].

Anti-Ro52/SSA antibodies in the fetus elicit an inflammatory response in the sinoatrial (SA) node, atrioventricular (AV) node, and the His bundle. This impairs impulse transmission, eventually leading to fibrosis and scarring of these conduction pathways, resulting in CHB and other conduction abnormalities characteristic of NLE [11].

In this case, considering the diagnosis of SLE and a history of a neonate with CHB, treatment with hydroxychloroquine (HCQ) was initiated. HCQ is the first drug shown to favorably modify fetal cardiac outcomes associated with anti-SSA/Ro antibodies. It reduces the recurrence of CHB in pregnancies exposed to anti-SSA/Ro antibodies by more than half and should be considered as a secondary preventive measure. Although HCQ was started at 17 weeks of gestation, later than the recommended timing in anti-SSA-positive pregnancies, it was initiated promptly after the diagnosis. Recent studies suggest that even mid-trimester initiation may help reduce the risk or severity of fetal cardiac injury [12].

This case also underscores the importance of early autoimmune evaluation in women with adverse pregnancy outcomes, particularly when the fetal complication is as specific as congenital heart block. In the patient’s previous pregnancy, no immunologic testing was performed, and subtle maternal symptoms—such as painless mucosal ulcers and intermittent joint swelling—were overlooked. These signs, although not alarming in isolation, were early indicators of an underlying autoimmune disorder. Earlier recognition and diagnosis might have altered the clinical course. The detection of anti-SSA antibodies and fulfillment of ACR/EULAR classification criteria [13] in the current pregnancy led to appropriate treatment and improved outcome. This highlights the need for thorough maternal assessment in the context of unexplained fetal loss or neonatal cardiac abnormalities [9, 14].

The diagnosis of SLE cannot be made solely based on serologic findings such as anti-SSA (Ro) positivity. Instead, the presence of systemic clinical manifestations is required in accordance with classification criteria. While obstetric history, such as prior stillbirth or neonatal cardiac complications, may raise clinical suspicion, it must be accompanied by relevant clinical signs to support the diagnosis and guide appropriate treatment. Additionally, accurate assessment and timely treatment play a vital role in reducing serious complications associated with this disease, especially during pregnancy. In this regard, medications such as HCQ, which can reduce fetal cardiac outcomes and prevent the recurrence of CHB, are of particular importance. It is important to note that a previous adverse fetal cardiac outcome, such as congenital heart block, does not definitively predict recurrence in future pregnancies. Favorable outcomes may occur spontaneously, even in the absence of or with minimal treatment. Therefore, while hydroxychloroquine may have contributed to the positive outcome in this case, a direct causal relationship cannot be assumed with certainty. Other modifying factors or chance may also have played a role [14]. Therefore, awareness of proper treatment methods and continuous follow-up in these patients is essential for maintaining the health of both the mother and the fetus.

## Electronic supplementary material

Below is the link to the electronic supplementary material.

Supplementary Material 1

## Data Availability

Data sharing is not applicable to this article as no datasets were generated or analyzed during the current study.

## Abbreviations

SLE: Systemic lupus erythematosus
ANA: Antinuclear antibody
NLE: Neonatal lupus erythematosus
CHB: Complete heart block
HCQ: Hydroxychloroquine
ACR/EULAR: American college of rheumatology / European league against rheumatism
